# The effect of 8‐week mindfulness counseling on sexual self‐efficacy of women suffering from human immunodeficiency syndrome: A randomized controlled trial in Iran

**DOI:** 10.1002/hsr2.1956

**Published:** 2024-03-11

**Authors:** Bahareh Sadeghian, Parvin Abedi, Najmeh Hamid, Elham Maraghi, Shahla Molavi

**Affiliations:** ^1^ Midwifery Department, Reproductive Health Promotion Research Center Ahvaz Jundishapur University of Medical Sciences Ahvaz Iran; ^2^ Midwifery Department, Menopause Andropause Research Center Ahvaz Jundishapur University of Medical Sciences Ahvaz Iran; ^3^ Counseling Department Shahid Chamran University of Ahvaz Ahvaz Iran; ^4^ Department of Biostatistics and Epidemiology, Faculty of Public Health Ahvaz Jundishapur University of Medical Sciences Ahvaz Iran; ^5^ Department of Health Psychology, School of Medicine Ahvaz Jundishapur University of Medical Sciences Ahvaz Iran

**Keywords:** counseling, HIV, human immunodeficiency syndrome, mindfulness, sexual self‐efficacy

## Abstract

**Background and Aims:**

Sexual self‐efficacy refers to a woman's belief that she can be sexually active and attractive for her sexual partner and has acceptable sexual function. One of the issues that may negatively affect sexual self‐efficacy is HIV infection. The present study aimed to evaluate the effect of 8 weeks of counseling based on mindfulness on sexual self‐efficacy of women affected with HIV.

**Methods:**

This was a randomized controlled trial involving 62 HIV‐positive women in their reproductive age. Women in the intervention group were further divided into four groups. Each group received eight sessions of counseling based on mindfulness, while the control group received no intervention. A demographic questionnaire and sexual self‐efficacy questionnaire were used to collect the data. The independent *t* test, *χ* ^2^, and repeated measure tests were used to analyze the data.

**Results:**

The mean change of total score of sexual self‐efficacy in Week 8 compared with before intervention was 37.04 (95% CI: 31.38–42.70, *p* < 0001) and in Week 12 compared with before intervention was 36.97 (95% CI: 31.59–42.34, *p* < 0.0001), while in the control group, it almost remained unchanged. The score of readiness for sexual relationship, sexual self‐stimulation, intimacy without sexual intercourse, and orgasm improved significantly 8 and 12 weeks after intervention (*p* < 0.0001), whereas no significant differences were observed in these dimensions in the control group.

**Conclusion:**

The results of this study showed that 8 weeks of counseling based on mindfulness could significantly improve all dimensions of sexual self‐efficacy and its total score in women affected with HIV. Thus, this method of counseling is recommended for HIV‐positive women.

## 1 INTRODUCTION

Human immunodeficiency virus (HIV) is an infectious disease in which the virus attacks the specific cells in the immune system known as cluster of differentiation 4 (CD4). CD4 are considered as helper cells as they boost the body's immune system response to infection.^1^ By the end of 2020, 37.7 million were living with HIV, and out of the 79.3 million people who were affected with this virus, 36.3 million died.^2^ The infection rate has been reported to be higher among women compared with men (19.3 million women as opposed to 16.7 million men were affected with HIV by the end of 2020).^1^

Statistics comprising 8‐year trend of HIV infection in Iran (from 2008 to 2016) indicated that the incidence of HIV increased from 4440 infected cases in 2008 to 4928 cases in 2016, with affected men outnumbering their women counterparts.^3^ According to a systematic review including 23 records and total participants of 199,855, the prevalence of HIV in Iran was 2.77% (95% CI: 1.96, 3.70).^4^ The routes of infection with the HIV virus are primarily through the drug injection and secondarily through sexual contact.^5^

In addition to the risk of death and disability, HIV infection may cause other diseases in women such as higher prevalence of cervical papilloma virus, higher rate of vaginitis due to *candida albicans*, and higher prevalence of adverse outcomes in pregnancy.^6^

HIV infection may negatively affect sexual function in women. According to Toorabally et al.,^7^ women living with HIV are more prone to have lower sexual function and having more sexual problems. One of the issues that may be negatively affected by HIV infection is sexual self‐efficacy. Sexual self‐efficacy refers to a woman's belief that she can be sexually active and attractive for her sexual partner and has acceptable sexual function.^8^ Low sexual self‐efficacy may negatively influence sexual function.^9^ Women who live with HIV infection and have higher self‐efficacy, are more likely to use protective methods against HIV transmission such as condoms.^10^ Sexual self‐efficacy can be enhanced by education. A study by Shokrani et al.^11^ reported that marital satisfaction significantly increased in women who received sexual self‐efficacy education. In a study on 451 HIV‐positive women to check the effect of cognitive behavioral stress management/expressive supportive therapy (CBSM) on stress and self‐efficacy, Jones et al.^12^ found that 10 weeks of counseling based on CBSM could significantly increase the self‐efficacy and reduce anxiety and depression of participants.

One of the counseling methods that can improve sexual self‐efficacy is mindfulness. Mindfulness therapy is usually used for increasing awareness about self and environment.^13^ Although a number of studies have shown that counseling methods such as mindfulness therapy can increase the quality of life of women living with HIV,^14^ there is not enough information regarding the effect of counseling based on mindfulness on the sexual self‐efficacy of women living with HIV. Therefore, the present study aimed to evaluate the effect of 8 weeks of counseling based on mindfulness on sexual self‐efficacy of women suffering from HIV.

## 2 MATERIALS AND METHODS

This was a parallel randomized controlled trial on 62 HIV‐positive women of reproductive age. The protocol of the study was approved by the Ethics Committee of Ahvaz Jundishapur University of Medical Sciences (Ref. No: IR.AJUMS.REC.1399.967). Also, the protocol was registered in the Iranian Registry for Randomized Clinical Trials (Ref. No: IRCT20210317050736N1, Registration date: 27/04/2021, URL: https://www.irct.ir/search/result?query=IRCT20210317050736N1).

The inclusion criteria were as follows: married women aged 18–45 with basic literacy and having at least one positive test for HIV such as rapid test or ELIZA, and a sexual self‐efficacy score less than 10. Women with the following characteristics were excluded from the study: using medications such as antidepressants and antihypertension that affect sexual function, having a male partner with sexual dysfunction or addiction to drugs or alcohol, having other medical and endocrine disorders such as diabetes and thyroid disorders based on the patient's medical record, and simultaneous attendance at counseling or other educational sessions to increase sexual self‐efficacy. All women provided written informed consent before data collection. The manuscript was prepared according to CONSORT guidelines.

### 2.1 Setting

This study was carried out in Ahvaz High Risk Behaviors Clinic. This clinic is a referral clinic for HIV‐positive patients in Khuzestan Province which is located in the Southwest of Iran and has a 4.711 million population according to the last national census in 2016. Ahvaz is the capital of Khuzestan province. Data collection was started in March 2021 and concluded in March 2022.

### 2.2 Randomization

Randomization was performed using block randomization with a block size of four and an allocation ratio of 1:1. The randomization was performed by a statistician. To implement allocation concealment, the codes dedicated to participants were placed in opaque envelops and kept with the clinic clerk. Therefore, neither the participants nor the researchers were aware of grouping. Due to the nature of the study blinding of participants or researchers in this study was not possible.

### 2.3 Sample size

Based on the objectives of the study and the methods of a previous study,^15^ and considering a power of 80%, *α* = 0.05, S1 = S2 = 12.3, and *d* = 9, the sample size was calculated according to the following formula:

$$n=\frac{{\left(Z_{1-\frac{\alpha }{2}}+Z_{1-\beta }\right)}^{2}(S_{1}^{2}+S_{2}^{2})}{{\left(d\right)}^{2}}.$$

The final sample size was calculated to be 30 in each group, and assuming 10% for attrition, it was increased to 33 in each group.

### 2.4 Intervention

Women in the intervention group were divided into four groups (three groups including eight participants and one involving nine). Each group received eight sessions of counseling based on mindfulness. We divided the intervention group into four groups, because counseling is done better in small groups. Later with emerging of COVID‐19, and considering social distancing, the groups reduced to four. All counseling contents were similar among groups.

The content of the sessions was as follows:

Session 1: The goals of sessions were presented, and the participants were introduced to each other. The importance of sexual self‐efficacy was explained. The participants were then instructed to do the mindful raisin eating exercise. Then they were requested to do a body scan meditation. Homework: doing physical exercise every day and mindful daily activities such as breathing and eating.

Session 2: This session involved physical exercise, homework review, and acceptance of thoughts and feelings exercise. Other activities in this session included recording pleasant events, 1–10‐min sitting meditation, visual and auditory meditation, 30–40‐min sitting meditating, and at the end of this exercise the body mindfulness is promoted. Homework: recording unpleasant events. Mindful walking and 3‐min breathing space practice three times a day.

Session 3: The participants were asked to identify the thoughts related to life events, examine unhealthy feelings caused by negative spontaneous thoughts, learn logical and realistic interpretation of unpleasant events, evaluate unhealthy feelings resulting from correcting thought errors, and be trained on recording events. The participants were instructed to be informed of their thoughts, and unpleasant feelings, how to have sex during treatment, and be trained on meditation. Homework: Meditation exercises, breathing techniques, and mindful raisin eating.

Session 4: In this session an educational lecture was given on stress and anxiety. Exercises in this session included those related to the present moment meditation and mindfulness. The participants were requested to do these exercises at home.

Session 5: In this session, an educational lecture was given to raise the participants' awareness and change their misconceptions using logical interpretation of events and correction of misconceptions, testing the relevant evidence in the real world, and having discussion with the group members.

Session 6: In this session, an educational lecture was given on utility analysis, practical utility analysis, teaching communication and problem‐solving skills, recognizing irrational beliefs and thoughts, cognitive reconstruction, and changing negative attitudes toward sexual issues.

Session 7: In this session, an educational lecture on the analysis of opposing beliefs, formation of opposing beliefs, teaching self‐punishment, self‐reward, strategies for developing a maintenance plan, learning cognitive skills, relaxation, imagination, and concentration, as well as how to deal with negative emotions.

Session 8: This session included a plan for follow‐up after treatment, evaluation of the positive results of the treatment plan and the participants' satisfaction with the treatment, implementation of the posttest, and closure of the program.

In this study, the control group received no intervention since there is no routine care about sexual matters in public health centers in Ahvaz, Iran. However, for ethical considerations, the control group attended one session on mindfulness and received a pamphlet for improving sexual self‐efficacy at the end of the intervention.

All counseling sessions were conducted by one of the researchers (B. S.) who was trained by a senior clinical psychologist, and all of these sessions were supervised by two of other research members (N. H. and S. M.).

Data collection for this study coincided with the COVID‐19 pandemic. Therefore, all safety precautions such as wearing a face mask and maintaining social distance were adhered in all counseling classes to reduce the possibility of disease transmission.

### 2.5 Outcomes

Sexual self‐efficacy that was measured at baseline, 8, and 12 weeks after intervention. The immediate measurement of sexual self‐efficacy showed the effect of intervention, while 4 weeks after completion of counseling the measurement showed the durability of counseling.^16^

### 2.6 Instruments

A demographic questionnaire and the Sexual Self‐Efficacy Questionnaire were used to collect data. The demographic questionnaire consisted of questions about age, age of the husband, education, education of the husband, economic status, occupation, how did they get infected and the method of contraception. The validity of this questionnaire was approved by content validity. The sexual self‐efficacy questionnaire was created by Bailes et al.^17^ This questionnaire has 37 questions that can evaluate a woman's self‐confidence in having sexual activity. Confidence ratings range from 10 (not at all sure) to 100 (very certain). If a question is not marked, the corresponding confidence score will be zero.

Rajabi et al. investigated the validity and reliability of the Persian version of this questionnaire. During psychometric evaluation, the number of questions in the questionnaire has been reduced to 28 questions. Questions 9, 10, 14, 22, 23, 24, 25, 28, 31 have been removed due to not being justified in Iranian culture. Exploratory factor analysis using Varimax rotation on 28 items initially identified eight factors with eigenvalues of more than 1, but four factors were distinguishable according to Scree test. These four factors explained 45.58% of total variance. Cronbach's *α* coefficients was *α* = 0.93 for the entire measure (28 items), ranging from 0.80 to 0.92 for the individual factors.^18^

### 2.7 Statistical analysis

The normality assumption of the continuous variables was examined using the Shapiro–Wilk *W* test. Categorical data were summarized as numbers (percentages). Continuous variables were reported as mean (SD, total range) or medians with interquartile. Two‐sided independent samples *t* test was used to compare the mean of age between two groups. The Pearson's *χ* ^2^ test was applied to test the difference in distribution of categorical variables between independent groups.

For examining the association between the type of intervention and changes in the sexual self‐efficacy score and its dimensions over time generalized estimating equation (GEE) models were used. The GEE models included two main effects (group and time) and the interaction of these effects. Time points in the analyses were at baseline and visits at Week 8 and Week 12. Pairwise comparisons following GEE analysis was done after Bonferroni correction. All tests were two‐sided and a *p* value < 0.05 was considered statistically significant. Data analyses were performed using the statistical software SPSS version 22 (IBM SPSS for Windows v22).

## 3 RESULTS

**Figure 1:**
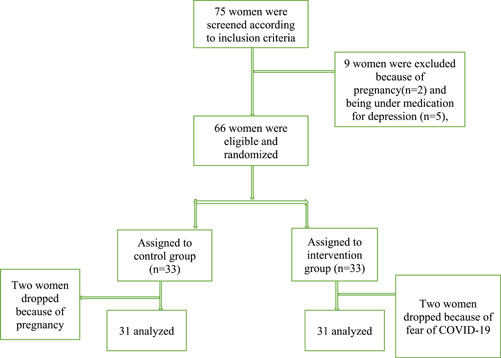
Diagram of recruitment and retention of participants in the study.

In this study, 66 HIV positive women were recruited of whom 62 completed the study. Two women in each group withdrew from the study. Reasons for withdrawal are presented in Figure 1. Table 1 demonstrates the demographic characteristics of the participants. The mean (SD) age of the participants was 34.41(5.53) and 36.25 (6.59) years in the intervention and control groups, respectively. Most of the women in the two groups were housewives, had primary or high school education, and had moderate economic status. Most of the participants had contracted HIV through sexual contact (self‐reported), and 23 (74.2%) and 16 (64.5%) of husbands in the intervention and control groups had been affected with HIV, respectively. Most of the participants in two groups used condom as the main contraception method (Table 1).

**Table 1:** Demographic characteristics of participants in the mindfulness counseling (MC) and control groups.

Characteristics	MC group	Control group
	*n* = 31	n = 31
Age; years		
Mean (SD, range)	34.41 (5.53, 25–46)	36.25 (6.59, 18–49)
Median (IQR)	33 (31–38)	37 (18–49)
Husband's age; years	41.35 (6.96, 30–60)	40.67 (6.32, 29–56)
Mean (SD, range)	41 (36–46)	39 (36–44)
Median (IQR)		

	*n* (%)	
Occupation		
Employee	2 (6.5)	6 (19.4)
Housewife	29 (93.5)	25 (80.6)
Education		
Primary	14 (45.2)	8 (28.8)
High school	6 (19.4)	7 (22.6)
Diploma	8 (25.8)	14 (45.2)
University degree	3 (9.7)	2 (6.5)
Husband's education		
Primary	3 (9.7)	2 (6.5)
High school	12 (38.7)	14 (45.2)
Diploma	14 (45.2)	13 (41.9)
University degree	2 (6.5)	2 (6.5)
Economic status		
Good	4 (12.9)	1 (3.2)
Moderate	22 (71)	19 (61.3)
Poor	5 (16.1)	11 (35.5)
HIV contract method		
Sexual contact	26 (83.9)	27 (87.1)
Other	5 (16.2)	4 (12.9)
Mode of delivery		
Normal	12 (38.7)	7 (22.6)
Cesarean section	18 (58.1)	22 (71)
Not having delivery	1 (3.2)	2 (6.5)
Husband affection with HIV		
Yes	23 (74.2)	16 (64.5)
No	8 (25.8)	11 (35.5)
Contraception's methods		
Condom	27 (87.1)	22 (71)
Hormonal methods	0 (0.0)	4 (13)
Tubal ligation	2 (6.5)	2 (6.5)
Withdrawal	2 (6.5)	3 (9.7)

Table 2 shows the scores of sexual self‐efficacy and its dimensions in the two groups before intervention, and in the 8th and 12th weeks of intervention. The total score of sexual self‐efficacy was improved from 7.05 ± 2.34 before intervention to 44.09 ± 11.01 in the 8th week and to 44.02 ± 10.41 in the 12th week of intervention (*p* < 0.0001) in the intervention group, while this score remained almost unchanged in the control group. Readiness for sexual relationship was improved from 6.56 ± 3.36 to 43.18 ± 12.01 in the intervention group after 12 weeks, while in the control group even decreased (*p* < 0.0001). The scores of sexual self‐stimulation improved in the intervention group from 10.87 ± 6.01 to 47.25 ± 11.92, while in the control group, it was improved slightly (*p* < 0.0001). Also, the score of intimacy without sexual intercourse was improved from 3.48 ± 3.68 to 37.41 ± 11.20 in the intervention group and in the control group the change was negligible (*p* < 0.0001). Finally, the score of orgasm improved from 8.14 ± 8.98 to 50.31 ± 12.24 in the intervention group and the changes in the control group was negligible.

**Table 2:** Estimated outcomes over time at baseline, Week 8, and Week 12 in the two study groups according to the generalized estimating equations (GEE) test.

Outcome	Baseline	Week 8	Week 12
*Total score of sexual self‐efficacy*			
MC group			
Mean (SD, range)	7.05 (2.34, 1.78–10.00)	44.09 (11.01, 25.35–64.30)	44.02 (10.41, 24.64–62.14)
Median (IQR)	7.85 (5.35–8.57)	43.92 (34.28–53.21)	45.71 (37.14–51.42)
Control group			
Mean (SD, range)	6.39 (2.52, 1.78–10.00)	6.44 (2.47, 1.78–10)	6.36 (2.52, 1.78–10.30)
Median (IQR)	6.42 (4.20–8.50)	6.40 (4.28–8.50)	6.07 (4.20–8.50)
*Readiness for sexual relationship*			
MC group			
Mean (SD, range)	6.56 (3.36, 0–13.84)	42.65 (11.81, 15.38–65.38)	47.25 (11.92, 21.66–70)
Median (IQR)	6.15 (3.84–8.46)	44.61 (33.84–50.00)	46.66 (38.33–56.66)
Control group			
Mean (SD, range)	6.14 (3.81, 0–13.84)	5.87 (4.01, 0–15.38)	8.97 (6.71, 0.00–23.33)
Median (IQR)	5.38 (3.07–8.46)	5.38 (2.30–8.46)	10 (1.66–15.00)
*Sexual self‐stimulation*			
MC group			
Mean (SD, range)	10.87 (6.01, 0–26.33)	46.88 (12.20, 26.66–70)	37.41 (11.20, 12–60)
Median (IQR)	11.66 (8.33–13.33)	46.66 (36.66–55.00)	36.00 (30.00–46.00)
Control group			
Mean (SD, range)	9.71 (6.21, 0–21.66)	9.32 (6.66, 0–21.66)	8.97 (6.71, 0.00–23.33)
Median (IQR)	11.66 (5.00–13.33)	10.00 (3.33–15.00)	10.00 (1.66–15.00)
*Intimacy without sexual intercourse*			
MC group			
Mean (SD, range)	3.48 (3.68, 0–14)	35.48 (11.48, 10–56)	37.41 (11.20, 12–60)
Median (IQR)	2 (0–6)	32.00 (26.00–44.00)	36.00 (30.00–46.00)
Control group			
Mean (SD, range)	3.09 (3.34, 0–10)	4.25 (4.21, 0–16)	2.58 (3.62, 0–16)
Median (IQR)	2 (0–6)	4 (0–8)	0 (0–4)
*Orgasm*			
MC group			
Mean (SD, range)	8.14 (8.98, 0–25)	49.19 (14.51, 20–80)	50.31 (12.24, 20–70)
Median (IQR)	5 (0–12.50)	52.50 (42.50–60.00)	50.00 (42.50–60.0)
Control group			
Mean (SD, range)	7.58 (8.59, 0–25)	8.30 (11.28, 0–50)	8.54 (11.41, 0–50)
Median (IQR)	5 (0–15)	5 (0–10)	5 (0–15)

*Note*: The *p* value for group × time interaction (based on the results of GEE analysis) was <0.0001 for all variables.

Abbreviations: IQR, interquartile range (25th–75th percentiles); MC, mindfulness counseling.

Table 3 shows Bonferroni‐corrected pairwise comparisons following GEE. As evident from this table, in all components of sexual self‐efficacy the differences between Week 8 and 0, Week 12 and 0 in the intervention group were significant, while the differences between Week 12 and 8 was not different. In the control group, the differences between Week 0, 8, and 12 was not significant.

**Table 3:** Bonferroni‐corrected pairwise comparisons following generalized estimating equations (GEE) analysis.

Outcome	MC group		Control group		Difference (MC minus control)	
	Mean (95% CI)	*p* Value*	Mean (95% CI)	*p* Value*	Mean (95% CI)	*p* Value*
*Total score*						
Week 8 minus Week 0	37.04 (31.38 to 42.70)	<0.0001	−0.61 (−2.37 to 1.15)	>0.99	37.65 (33.74 to 41.55)	<0.0001
Week 12 minus Week 0	36.97 (31.59 to 42.34)	<0.0001	−0.03 (−0.17 to 0.11)	>0.99	37.00 (33.22 to 40.78)	<0.0001
Week 12 minus Week 8	−0.07 (−2.01 to 1.87)	>0.99	−0.07 (−0.28 to 0.12)	>0.99	0.00 (−1.30 to 1.31)	0.994
*Readiness*						
Week 8 minus Week 0	36.08 (29.66 to 42.49)	<0.0001	−0.27 (−1.82 to 1.28)	>0.99	36.35 (31.94 to 40.76)	<0.0001
Week 12 minus Week 0	36.61 (29.99 to 43.24)	<0.0001	0.42 (−1.73 to 2.58)	>0.99	36.19 (31.54 to 40.85)	<0.0001
Week 12 minus Week 8	0.53 (−1.36 to 2.43)	>0.99	0.69 (−0.99 to 2.38)	>0.99	−0.16 (−1.85 to 1.53)	0.854
*Self‐stimulation*						
Week 8 minus Week 0	36.01 (29.75 to 42.28)	<0.0001	−0.39 (−2.24 to 1.46)	>0.99	36.40 (32.04 to 40.77)	<0.0001
Week 12 minus Week 0	36.38 (30.85 to 41.90)	<0.0001	−0.74 (−3.06 to 1.58)	>0.99	37.12 (33.12 to 41.12)	<0.0001
Week 12 minus Week 8	0.36 (−2.55 to 3.28)	>0.99	−0.34 (−2.43 to 1.73)	>0.99	0.71 (−1.68 to 3.10)	0.560
Week 8 minus Week 0	32.00 (25.77 to 38.22)	<0.0001	1.16 (−0.85 to 3.17)	>0.99	30.83 (26.47 to 35.20)	<0.0001
Week 12 minus Week 0	33.93 (27.70 to 40.16)	<0.0001	−0.51 (−2.75 to 1.72)	>0.99	34.45 (30.03 to 38.87)	<0.0001
Week 12 minus Week 8	1.93 (−1.45 to 5.32)	>0.99	−1.67 (−3.59 to 0.23)	>0.99	3.61 (1.01 to 6.21)	0.006
*Orgasm*						
Week 8 minus Week 0	41.04 (32.71 to 49.38)	<0.0001	0.72 (−5.23 to 6.68)	>0.99	40.32 (33.48 to 47.16)	<0.0001
Week 12 minus Week 0	42.17 (34.95 to 49.39)	<0.0001	0.96 (−5.97 to 7.90)	>0.99	41.20 (34.52 to 47.89)	<0.0001
Week 12 minus Week 8	1.12 (−3.47 to 5.72)	>0.99	0.24 (−3.82 to 4.30)	>0.99	0.88 (−3.21 to 4.98)	0.673

Abbreviations: CI, confidence interval; GEE, generalized estimating equations; MC, mindfulness counseling.

* *p* Values from contrasts of MC group versus control group in a GEE model of outcome as a function of group, time, and group × time.

## 4 DISCUSSION

This study was designed to evaluate the effect of counseling based on mindfulness on sexual self‐efficacy of HIV‐positive women. The results of this study showed that the scores of all dimensions of sexual self‐efficacy, as well as its total score in the two groups were low before intervention, but 8 and 12 weeks after intervention these scores improved significantly in the intervention group compared with the control group. Although we could not find any study to specifically assess the effect of mindfulness counseling on sexual self‐efficacy in women with HIV, a number of other studies, consistent with ours, have shown the positive effect of counseling on sexual self‐efficacy of women in reproductive age. For example, Ismaeilzadeh et al.^19^ found that 8 weeks of counseling according to mindfulness cognitive behavioral therapy could significantly improve sexual self‐efficacy among reproductive‐aged women. Mohammadizadeh et al.^20^ reported significant improvement of sexual self‐efficacy in women with breast cancer after 8 weeks of counseling based on mindfulness.

A study by Viseskul et al.^21^ on 92 HIV‐positive Thai youth showed the sexual self‐efficacy of their participants was low and they needed intervention to improve their sexual self‐efficacy.

Despite their low sexual self‐efficacy, HIV‐positive people have low general self‐efficacy, which may prevent them from HIV testing uptake^22^ or may be associated with unprotected sex and less HIV testing.^23^ Khosravy et al. in their study found that self‐efficacy and higher education level are important factors to predict HIV prevention behaviors.^24^ Therefore, self‐efficacy is an important factor as far as risk reduction among HIV‐positive patients is concerned.^25^ Hassanshahi et al. in their study on 84 women with high‐risk behavior found that an educational intervention program based on the Information‐Motivation‐Behavioral Skills could significantly increase sexual self‐efficacy of women with high‐risk behavior.^26^ Confirming the results of Hassanshahi et al. another study by Richer et al. showed that HIV knowledge and sexual self‐efficacy could predict the preventive behaviors regarding HIV.^27^

In addition to their low sexual self‐efficacy, Iranian HIV‐positive women, especially those whose husbands are negative for the disease, face many sexual problems. For example, Rostamkhani et al.^28^ found that around 70% of HIV‐positive Iranian women in the reproductive age had sexual dysfunction. By the same token, also, Nedjat et al.^29^ reported that the sexual and reproductive needs of Iranian women with HIV were not met by the Iranian health system. Finally, these women have been found to have little knowledge with regard to preventing the transmission of the disease to others and using protective sexual behaviors.^30^

### 4.1 Strengths and limitations of study

This is the first study to evaluate the effect of counseling based on mindfulness on sexual self‐efficacy of HIV‐positive Iranian women. Despite its strengths, this study has some limitations. First, talking about sexual issues is taboo in the Iranian culture and this may have affected the results. Second, the data collection was done during COVID‐19 pandemic, and this may have caused fear and anxiety and affected the participants' mental state. Third, in this study, we did not invite husbands to attend classes, and since spousal cooperation is required to increase sexual self‐efficacy, this may have affected our results. Fourth, although we checked some confounding variables, there are other confounding variables that affect sexual self‐efficacy that did not measure in this study, and this may cause bias. And finally, this study conducted in one high‐risk behavioral center in Ahvaz, and this limit the generalizability of this study.

## 5 CONCLUSION

The results of this study showed that 8 weeks of counseling based on mindfulness could significantly improve all dimensions of sexual self‐efficacy as well as its total score in women affected with HIV. Therefore, this method of counseling is recommended for HIV‐positive women.

## AUTHOR CONTRIBUTIONS

**Bahareh Sadeghian**: Conceptualization; data curation; formal analysis; methodology; software; writing—review and editing. **Parvin Abedi**: Conceptualization; formal analysis; funding acquisition; investigation; methodology; software; supervision; validation; writing—original draft; writing—review and editing. **Najmeh Hamid**: Conceptualization; methodology; software; supervision; validation; writing—review and editing. **Elham Maraghi**: Conceptualization; investigation; methodology; software; validation; visualization; writing—review and editing. **Shahla Molavi**: Conceptualization; methodology; supervision; validation; visualization; writing—review and editing.

## CONFLICT OF INTEREST STATEMENT

The authors declare no conflict of interest.

## TRANSPARENCY STATEMENT

The lead author Parvin Abedi affirms that this manuscript is an honest, accurate, and transparent account of the study being reported; that no important aspects of the study have been omitted; and that any discrepancies from the study as planned (and, if relevant, registered) have been explained.

## Data Availability

Data of this study will be available upon the reasonable request from corresponding author.
